# CanAssist Breast Impacting Clinical Treatment Decisions in Early-Stage HR+ Breast Cancer Patients: Indian Scenario

**DOI:** 10.1007/s13193-019-01014-4

**Published:** 2019-12-09

**Authors:** Satish Sankaran, Jyoti Bajpai Dikshit, Chandra Prakash SV, SE Mallikarjuna, SP Somashekhar, Shekhar Patil, Rajeev Kumar, Krishna Prasad, Dinesh Shet, Manjiri M. Bakre

**Affiliations:** 1 OncoStem Diagnostics Private Limited, 4, Raja Ram Mohan Roy Road, Aanand Towers, 2nd Floor, Bangalore, Karnataka 560027 India; 2 Manipal Hospital, Bangalore, India; 3 Sri Shankara Cancer Hospital and Research Center, Bangalore, India; 4 HCG, Bangalore, India; 5 Rajiv Gandhi Cancer Institute and Research Center, New Delhi, India; 6 Mangalore Institute of Oncology, Manipal, India; 7 Father Muller Medical College, Mangalore, India

**Keywords:** CanAssist-Breast, Decision impact, Indian patients, Prognostic, Early-stage breast cancer

## Abstract

CanAssist Breast (CAB) has thus far been validated on a retrospective cohort of 1123 patients who are mostly Indians. Distant metastasis–free survival (DMFS) of more than 95% was observed with significant separation (*P* < 0.0001) between low-risk and high-risk groups. In this study, we demonstrate the usefulness of CAB in guiding physicians to assess risk of cancer recurrence and to make informed treatment decisions for patients. Of more than 500 patients who have undergone CAB test, detailed analysis of 455 patients who were treated based on CAB-based risk predictions by more than 140 doctors across India is presented here. Majority of patients tested had node negative, T2, and grade 2 disease. Age and luminal subtypes did not affect the performance of CAB. On comparison with Adjuvant! Online (AOL), CAB categorized twice the number of patients into low risk indicating potential of overtreatment by AOL-based risk categorization. We assessed the impact of CAB testing on treatment decisions for 254 patients and observed that 92% low-risk patients were not given chemotherapy. Overall, we observed that 88% patients were either given or not given chemotherapy based on whether they were stratified as high risk or low risk for distant recurrence respectively. Based on these results, we conclude that CAB has been accepted by physicians to make treatment planning and provides a cost-effective alternative to other similar multigene prognostic tests currently available.

## Background

Among all cancers, breast cancer occupies the top position in Indian women [1]. Based on projections, breast cancer patient numbers could reach 1.9 million by 2020 [2]. Mortality to incidence rate (MIR) is a useful indicator of 5-year survival across various cancers [3]. In a study stratifying MIR for breast cancer with Human Development Index (HDI) which is a composite measure of education, income, and life expectancy, MIR for a medium HDI country like India in 2016 was found to be 0.5 compared with 0.19 for a very high HDI country like the USA [4]. Besides economic reasons, Indian patients also present with more aggressive form of the disease, with an earlier age at onset and higher tumor stage and node positivity. Even with the presence of high risk factors, not every patient might benefit from chemotherapy and the challenge is in identifying these patients accurately.

In the past 60 years, since the first cancer patient enrolled in a randomized clinical trial conducted by the National Surgical Adjuvant Breast and Bowel Project (NSABP) [5], there has been constant improvement in the effective treatment and surgical management of breast cancer patients. Starting with a combination of cyclophosphamide, methotrexate, and 5-flurouracil (CMF) in the 1950s, several new drugs (anthracyclines and taxanes) and drug regimens have since evolved [6], in addition to hormone therapy, for hormone receptor–positive breast cancer. While the efficacy improved in some cases, these drugs also produced unwanted side effects like cardiotoxicity and decrease in blood cell counts.

Optimal treatment helps in minimizing mortality and morbidity associated with the disease. Treating every patient with the most aggressive form of treatment may not always be productive, even if the patient does not have existing comorbidities. The Cochrane review compiled data from 14 randomized clinical trials involving 5600 women. There was quality data to show that high doses of chemotherapy did not improve survival in early-stage breast cancer patients [7]. The harmful side effects of chemotherapy are well known and balancing the harm to benefit from chemotherapy would improve the quality of life of patients. With the advent of multigene prognostic tests, studies have shown 70% early-stage hormone receptor–positive, node-negative breast cancer patients benefit from chemotherapy [8].

Some of the commercially available prognostic tests include Oncotype DX (ODX) [9], MammaPrint [10], Prosigna [11], EndoPredict [12], Breast Cancer Index (BCI) [13], and CanAssist Breast (CAB) [14]. All these tests query different gene/protein markers and use different testing methodologies (Table 1) but have been shown to predict risk of recurrence with more than 95% accuracy.

**Table 1:** Summary of commercially available prognostic tests

	Oncotype DX	MammaPrint	Prosigna	EndoPredict	Breast Cancer Index	CanAssist Breast
No. of genes used	21	70	50	8	7	5 biomarkers
Proliferation genes included	Yes	Yes	Yes	Yes	No	No
Method	qPCR	DNA microarray	NanoString	qPCR	qPCR	IHC
Clinical parameters	No	No	No	Tumor size, node status	No	Tumor size, node status, and tumor grade
Presence of intermediate zone	Yes	No	Yes	No	No	No
Prediction of chemotherapy benefit	Yes	No	No	No	No	Yes

Of all these tests, CAB is the only test that has been developed and validated on a mixed cohort of Asian (Indian) and Caucasian patients in a 3:1 proportion [15] and extensively validated for analytical performance [16]. CAB is an immunohistochemistry (IHC)-based test that assesses risk of cancer recurrence at a distant site within 5 years from diagnosis. It quantifies protein expression levels of a combination of 5 unique non-proliferative biomarkers (CD44, Pan-Cadherin, N-Cadherin, ABCC4, and ABCC11). CAB markers are involved in diverse cancer signaling pathways that regulate cancer metastasis and drug resistance. The IHC data from the biomarkers are combined with three clinical parameters, tumor size (T), node status (N), and tumor grade, to generate a low- or a high-risk score for every patient using a machine learning–based statistical model.

The Indian Council of Medical Research (ICMR) does not include tests like Oncotype DX and MammaPrint in their recommendations for treatment decision-making due to the lack of data on Indian patients (https://www.icmr.nic.in/sites/default/files/guidelines/Breast_Cancer.pdf). The critical point of development and validation on Indian patients makes CanAssist-Breast a good choice for Indian patients.

CanAssist Breast test has been used for treatment planning for patients since 2016. The aims of the present study are to briefly summarize the retrospective validation data about CAB and analyze the clinical scenarios for recommending this test and correlation of risk stratification by CAB to treatment decisions by referring physicians.

## Methods

### Patient Selection

For the retrospective study, we obtained post-surgical tumor samples in the form of formalin-fixed paraffin-embedded (FFPE) blocks from 1123 early-stage breast cancer patients. Patient consent, patient information, and treatment follow-up details such as age, year of diagnosis, type of surgery, tumor size and grade, hormone receptor status, node status, treatment regimen, date of recurrence or last visit, or death were obtained from the treating hospitals. All patients had hormone receptor–positive disease. TNBC patients were excluded from the study. The patients were staged based on the AJCC 7th edition staging system. Patients with tumors with stage I (T1N0) and stage II (T1N1, T2N0, T2N1, T3N0) were considered as early stage. Patients with a minimum of 5-year follow-up were included and this requirement was waived off only in patients with a recurrence at a distant site within the 5-year period.

For the decision impact study, patients who were recommended testing by CanAssist Breast by their treating physician between 2016 and May 2019 were included in this study. Patient consent was taken as part of test requisition form. Clinical information like node, tumor size, and tumor grade was obtained from pathology reports obtained from the patients along with the test requisition form. ER/PR status was included as a mandatory requirement for acceptance for CanAssist Breast testing and the report was provided by the patient or hospital from where the cases were referred by. Ki67, wherever available along with the ER/PR reports, was used for luminal classification in this study. Referring physician name and patient age were obtained from the test requisition form. Luminal subtyping was performed as per St. Gallen’s recommendations [17].

### Study Design

A total of 455 patients who were recommended CAB for treatment planning were included for various subgroup analyses in this study. To assess region-wise distribution of prescribing physicians from India, five different zones were considered. These included North (Delhi, Uttar Pradesh, Haryana, and Rajasthan), Central (Madhya Pradesh), West (Maharashtra, Gujarat, and Rajasthan), East (West Begal), and South (Karnataka, Kerala, Andhra Pradesh, and Tamil Nadu). Prescriptions were also received from neighboring countries of the Indian subcontinent comprising of Sri Lanka, Pakistan, and Bangladesh which were considered as “outside India” for analysis. Information on usefulness of CAB-based risk stratification for treatment planning was obtained from prescribing physicians via email and personal visits. Data was collected and analyzed to assess if patients stratified as either low risk or high risk for recurrence by CAB were given chemotherapy or not for patients who were prescribed CAB. This follow-up information was obtained for 254 patients.

### Statistical Analysis

Kaplan-Meier survival curve analysis was performed using GraphPad version 8. Distant metastasis–free survival (DMFS) was calculated for CAB high-risk versus low-risk patients. *P* values were computed using log-rank two-sided test at 0.05 significance. DMFS is the time interval between the date of diagnosis of cancer and the last date of follow-up in case of no event/recurrence with a minimum period of 5 years.

MedCalc (https://www.medcalc.org/calc/comparison_of_proportions.php) was used to calculate statistical significance of proportions. *P* values less than or equal to 0.05 were considered to be statistically significant.

### CanAssist Breast Testing

Testing was performed on FFPE blocks. The CAB was performed as described previously [14, 15]. Briefly, immunohistochemistry was performed for the 5 CAB biomarkers at the CAP-accredited central reference OncoStem lab at Bangalore, India. The IHC grading information was incorporated along with the 3 clinical parameters (tumor size, tumor grade, and nodal status) as obtained from the treating hospitals to calculate CAB risk scores on a scale of 0–100. A cutoff of 15.5 is used to classify patients into low risk (score ≤ 15.5) or high risk (score > 15.5) for distant recurrence [14].

### Adjuvant! Online–Based Risk Categorization

The modified Adjuvant! Online (ver8) criteria as described in the MINDACT trial [18] was used for assigning risk categories based on tumor grade, node status, and tumor size (Table 2). A total of 430 cases for which the exact T size and number of metastatic nodes data were available were used in this concordance analysis.

**Table 2:** Criteria used for clinical risk classification

Grade	Nodal status	Tumor size	Clinical risk
Well differentiated (grade 1)	N0	≤ 3 cm	C-Low
		3.1–5 cm	C-High
	N1 (1–3 positive nodes)	≤ 2 cm	C-Low
		2.1–5 cm	C-High
Moderately differentiated (grade 2)	N0	≤ 2 cm	C-Low
		2.1–5 cm	C-High
	N1 (1–3 positive nodes)	Any size	C-High
Poorly differentiated (grade 3)	N0	≤ 1 cm	C-Low
		1.1–5 cm	C-High
	N1 (1–3 positive nodes)	Any size	C-High

## Results

### Performance of CAB on Retrospective Cohort

**Fig. 1:**
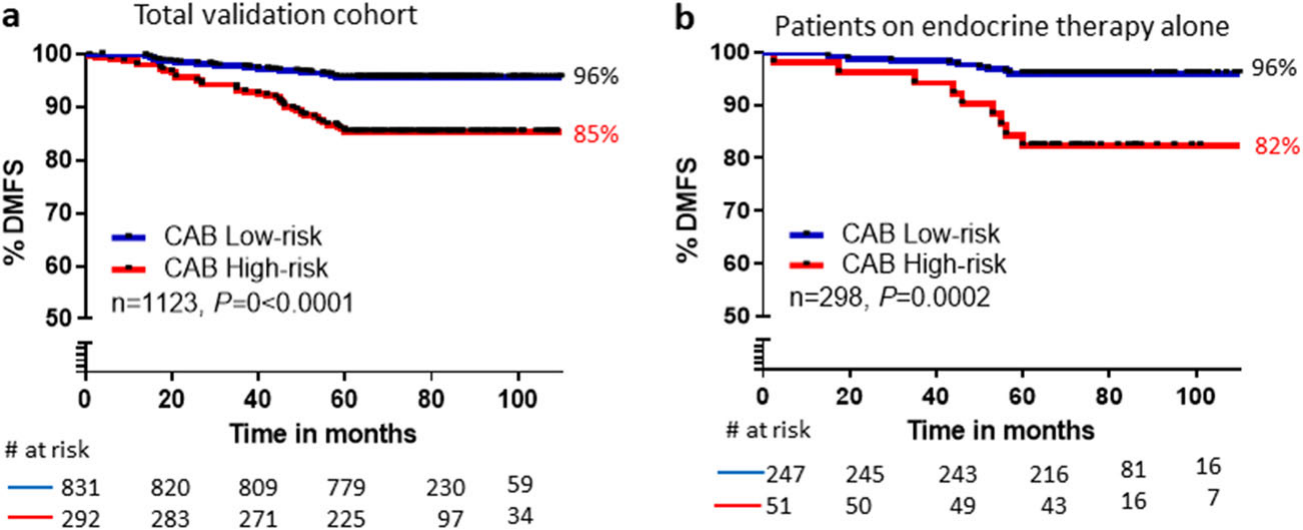
Performance of CAB. **a** Survival (KM) curve using CAB-based risk categorization on a retrospective mixed cohort of chemotherapy-treated and chemotherapy-naïve (endocrine therapy alone treated) patients. **b** Survival analysis using CAB-based categorization with chemotherapy-naïve patient cohort

Retrospective patients (*n* = 1123) were dichotomized into low- and high-risk groups by CAB. There was 11% difference in the DMFS between low- and high-risk groups demonstrating statistically significant (*P* < 0.0001) separation between the two groups (Fig. 1a). Since this cohort had a mix of patients with and without chemotherapy treatment, we further performed survival analysis on patients who were treated with endocrine therapy alone (*n* = 298). This was to exclude any confounding effect of chemotherapy in the mixed cohort. The separation between the low- and high-risk groups was significant (*P* = 0.0002) for this subgroup as well (Fig. 1b) with a clear difference in DMFS of 14% between the two risk groups. The DMFS for both the mixed and chemotherapy-naïve subgroups was > 95% in the low-risk group.

### Evaluation of Various Risk Factors in the Study Cohort in the Prospective Cohort

Upon successful completion of clinical, analytical validation of CanAssist-Breast and upon getting the appropriate regulatory accreditations, CAB was launched in the market from the middle of 2016. Until date, over 500 patients have availed CAB in the Indian subcontinent. Analysis has been shown in the subsequent sections on the prospective cohort of 455 patients.

As shown in the retrospective clinical validation cohort data [15], we had observed that most patients had T2 tumors with grade 2 disease and node-negative disease [15]. We wanted to evaluate if this was the case with prospective patient cohort who were prescribed CAB. We therefore performed subgroup analysis based on node status, tumor size, tumor grade, and age. There was a high proportion of patients with N0 (82.4%), T2 (58.4%), and grade 2 (65.3%) disease (Table 3). Thus, clinical characteristics were predominantly similar to that of the retrospective cohort used for test validation.

**Table 3:** Cohorts’ description (*n* = 455). For T size, *n* = 448; luminal subtyping, 265 patients for whom Ki67 status was known were considered

Parameter	Number of patients (%)
T1	178 (39.6)
T2	262 (58.4)
T3	9 (2.0)
N0	375 (82.4)
N+	80 (17.6)
G1	90 (19.8)
G2	297 (65.3)
G3	68 (14.9)
Luminal A	83 (31.3)
Luminal B	182 (68.7)
Age < 40 years	23 (5.0)
Age 41–60 years	252 (55.4)
Age > 61 years	180 (39.6)

In the node-positive group of the prospective cohort, 94% of patients presented with N1 disease. Sixty-five percent of the patients with N1 disease were single-node positive, 16% were double-node positive, and 13% were triple-node positive.

Based on the tumor sizes, 92% of T1 patients had a tumor size of ≥ 1 cm; 85% and 27% of T2 patients had tumor sizes between 2–3 and 3–4 cm respectively; 1.8% of patients had tumor size > 5 cm. Percentage of grades 1 and 3 were 19.8 and 14.9% respectively.

55.4% of patients were in the age group of 41–60 years and 5% patients had an age at onset of ≤ 40 years. 68.7% of patients were of the luminal B subtype (Table 3).

### Clinical Factors Influencing CAB Risk Categorization

We further evaluated the effect of the clinical factors on CAB-based risk categorization. Luminal subtype and age had no significant effect on proportions of low-risk stratified patients by CAB, while the influence of tumor size, node, and tumor grade was significant (Table 4). We also evaluated the performance of the test across patients from two geographically distinct zones (North and South) of India and found that the proportions of low- and high-risk patients was not significantly different across these two patient subgroups (*P* = 0.8), and to the total pooled subgroup (*P* = 0.7 for South vs total; *P* = 0.6 for North vs total).

**Table 4:** Significance of low-risk proportions across various disease parameters

Parameter	% low risk	*P* value
Luminal A	70	*P* = 0.5
Luminal B	69	
T1	83	*P* < 0.0001
T2+T3	61	
N0	74	*P* = 0.0004
N+	54	
G1	89	*P* < 0.0001
G2+G3	65	
< 40 years	74	*P* = 0.7
> 40 years	70	

### Comparison of CAB with Adjuvant! Online on Risk Categorization

**Fig. 2:**
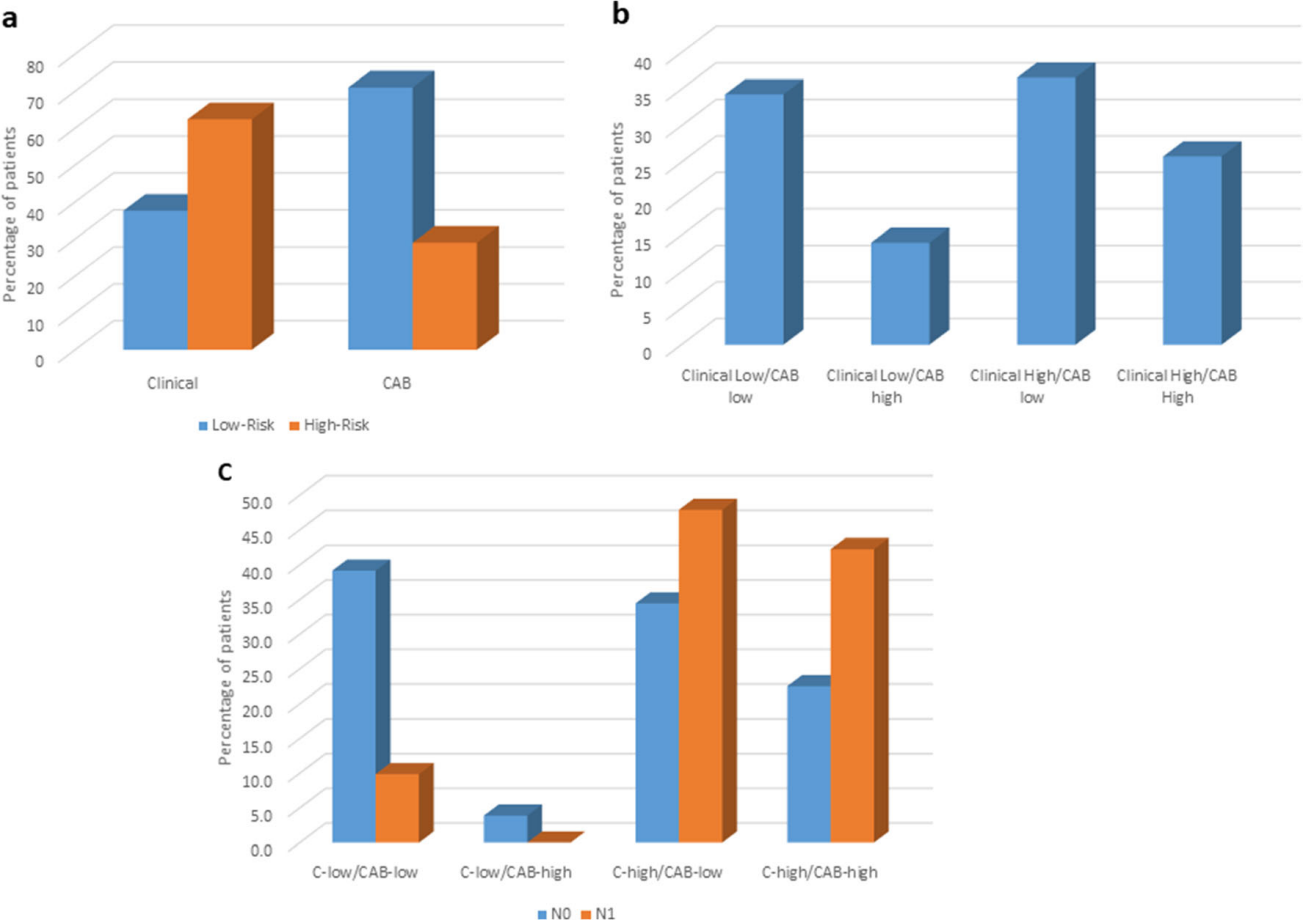
Comparison of risk categorization by CAB vs Adjuvant! Online. **a** Proportions of high- and low-risk categorization by the two tests. **b** Differential risk categorization by CAB vs Adjuvant! Online irrespective of node status. **c** Differential risk categorization by CAB vs Adjuvant! Online based on node status

In the scenario where patients cannot afford expensive prognostic tests, physicians tend to decide on therapy options based on various clinical parameters and freely available online predictive tools like Predict [19], NPI [20], and Adjuvant! Online (AOL) [21]. While NPI and Predict provide estimate of overall survival, Adjuvant! Online provides risk of recurrence and potential response to chemotherapy. Since both CAB and Adjuvant! Online aid in making treatment decisions, we compared the performance of CAB with AOL that predicts risk based on clinical parameters alone. For those patients for whom we had information of the exact size of the tumor (*n* = 430), we compared the risk categorization by AOL vs CAB. AOL (clinical) categorized 62.4% patients into high risk as compared with 29% by CAB (Fig. 2a). CAB categorized 36.6% of AOL (clinical) high-risk patients into low risk (Fig. 2b). CAB categorized 34.4% N0 and 47.9% of N+ AOL (clinical) high-risk patients into low risk (Fig. 2c).

### Physician Considerations for Testing

**Fig. 3:**
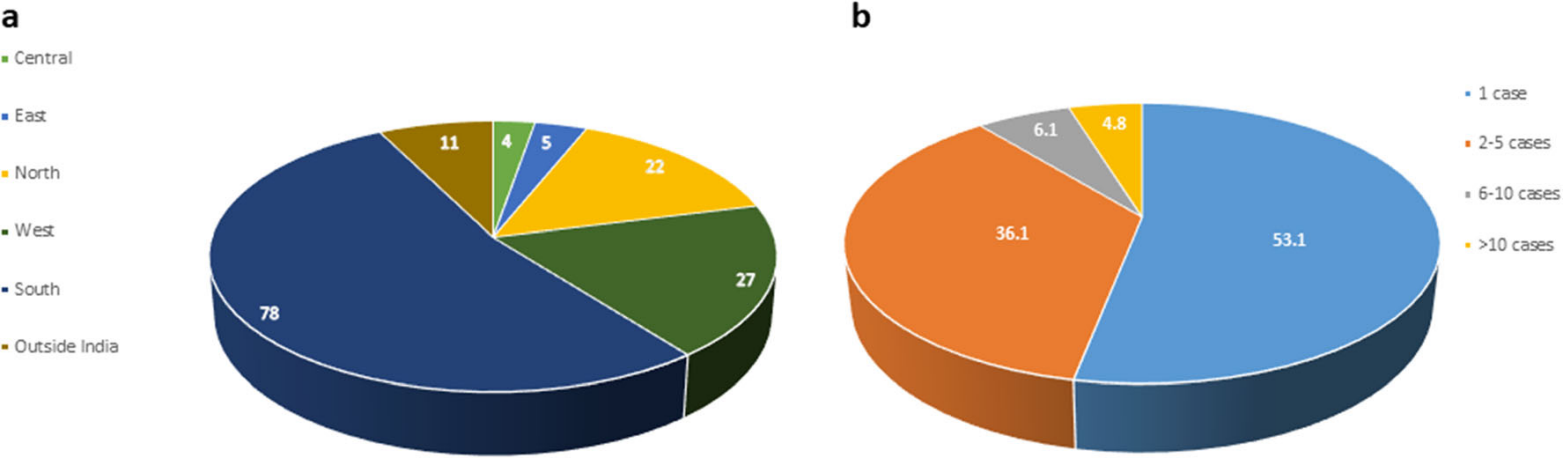
Physician prescriptions. **a** Geographical distribution of prescribers of CAB in India and outside India (*n* = 440). **b** Percentage of physician prescriptions across based on number of cases prescribed

The tests were prescribed by 147 physicians from all across India (North, South, Central, East, and West) and from neighboring countries (Fig. 3a). While 47% of physicians prescribed the test for more than one patient with 11% of physicians prescribing > 6 patients for CAB testing (Fig. 3b). Next, we wanted to assess the reasons for prescribing CAB by physicians to their patients. From the survey conducted by us, the major reasons were (a) presentation with high-risk clinical features like large tumors or node positivity, (b) to assess if patients with luminal A disease would benefit from chemotherapy, (c) young age of patients who want to avoid side effects of chemotherapy, (d) old aged patients who might be spared chemotherapy if possible, and (e) comorbidities in the patient that might require evaluating the benefit of chemotherapy over its effect that would have a bearing on the quality of life. Across all these reasons, the main rationale for prescription for all the physicians was to avoid chemotherapy, if possible.

### Physician’s Consideration of Treatment Change Based on CAB-Based Stratification

The goal of risk of recurrence prediction tests is to help guide the physician in the decision-making of patient’s treatment planning. Thus, it was important to assess how CAB helped in guiding the treatment decision. CAB is not a stand-alone test to determine inclusion of chemotherapy into the treatment regimen and clinicians do rely on other patient-specific historical information, comorbidities to arrive at this conclusion. However, since low-risk patients would potentially not benefit from chemotherapy while high-risk patients would benefit from chemotherapy, data was analyzed for both low-risk and high-risk patients to assess if they did or did not receive chemotherapy treatment. We observed that 88% (*n* = 254) of patients received chemotherapy or not based on whether they were CAB high or low risk respectively. (Table 5). Interestingly, 92% of patients (159/173) stratified as low risk for recurrence by CAB did not receive chemotherapy as compared with 80% (65/81) of high-risk patients who received chemotherapy. Of the low-risk patients who received chemotherapy, it was mostly the patient’s decision to opt for chemotherapy.The prime reasons for high-risk patients who did not receive chemotherapy were older age and patient’s preference.

**Table 5:** Treatment decsions based on CAB-based risk categorization

Risk category by CAB	No. of patients	Chemotherapy given	Chemotherapy not given	Percentage of patients
Low risk	173	14	159	92% did not receive chemotherapy
High risk	81	65	16	80% received chemotherapy
Total	254	159 + 65 = 224		88% received chemotherapy if they were high risk and no chemotherapy if they were low risk

## Discussion

The incidence of breast cancer in India is much lower than that in the West but nonetheless is the most common cancer in Indian women [1]. Majority of patients present the disease at stages II and III with node negativity [22–24]. The median age at onset is < 60 years, with majority of patients presenting with T2 tumors. In early-stage patients, majority were diagnosed at stage II with N0 node status [22]. More than 80% were of the invasive ductal carcinoma (IDC) morphology [23] with 20–33% hormone receptor positivity [22, 23]. The retrospective cohort used to validate CAB reflect a very similar proportions of clinical features with a majority of T2 tumors (65%), median age at onset of < 60 years, and node negative (57%) indicating that the validation cohort used for CAB [15] is a true representation of the breast cancer disease characteristics reported in India.

CAB is robust in risk classification and is not affected by age at onset, luminal subtypes, or geographical locations. This could be attributed to the type of patient samples used for development and validation of the test. CAB has been shown to perform well across all age groups, irrespective of menopausal status. It is also important to note that the set of biomarkers used in this test are not part of the ER/PR signaling or proliferative pathways, unlike other tests like Oncotype DX and EndoPredict. This could explain why this test does not get affected by the luminal subtypes which mainly are determined by the expression levels of ER/PR and Ki67. On similar lines, we have also shown that CAB performs better than IHC4- and Ki67-based prognostication [15] on a retrospective cohort with correlation to outcome.

There are multiple free online tools, like Predict and NPI, available that are often used by physicians as substitutes for prognostic tests. It is however important to ascertain the limitations of these tools before using them to decide patient treatment. Both NPI and Predict provide overall survival information and may not be accurate in exactly predicting benefit from chemotherapy treatment. NPI has been shown to provide suboptimal prognosis in patients who are < 40 years and significantly underestimated overall survival in patients aged between 55 and 60 years [25]. Despite validation of Predict in European, US, and Asian patients, it has been shown to work accurately only for Western patients within the age group of 50–65 years [26, 27]. In this study, we compared the performance of Adjuvant! Online with CAB for risk categorization. We used the modified version of Adjuvant! Online (AOL) as described in the MINDACT study to stratify patients into clinical low- and high-risk categories. CAB categorizes more patients into low risk as compared with AOL as observed in both node-negative and node-positive subgroups. It is interesting to note that CAB identified patients who might benefit from chemotherapy in the clinical low-risk category with node-negative disease. On the other hand, 34.4% and 47.9% AOL high-risk patients with node-positive and node-negative disease, respectively, could avoid chemotherapy as CAB categorized them as low risk. Based on this AOL and CAB comparison data, it is evident that clinical parameters alone are not significant in predicting risk of recurrence and the tumor biology contribution from the CAB biomarkers adds great value in more accurate risk prediction.

St. Gallen’s 2013 guidelines suggest that only patients with luminal A and grade 3 disease could be given chemotherapy. Data from this study suggests that 17 out of the 73 (23%) who were luminal A with grade 1 or 2 stratified as high risk by CAB would not have received chemotherapy as per these guidelines leading to potential undertreatment. Two patients out of 10 who had luminal A grade 3 disease were stratified by CAB as low risk and thus could avoid chemotherapy. On the other hand, all luminal B patients are eligible for chemotherapy as per these guidelines. We observed that 69% (126 out of 182) of patients with luminal B disease were low risk by CAB and could avoid chemotherapy. Of these, 111 patients had node-negative disease. The data highlights the usefulness of CAB in avoiding both under- and overtreatment of patients based on prognostic tests. It is noteworthy that the clinical utility of prognostic tests is recognized by international guidelines like AJCC (8th edition, 2018) and St. Gallen’s recommendations (2013) as useful tools for disease staging and to decide treatment plans respectively.

Tests like MammaPrint, ODX, and EndoPredict have been developed and validated on non-Asian populations. Multiple studies have shown that the clinical characteristics of breast cancer in Asians (mostly Chinese) are very different from those from the West [28, 29]. Asians presented with much later stages of disease (stage II or above), larger tumors, and a much lower age at onset than their Western counterparts. It is thus important to evaluate the performance of any test in the appropriate population before including them in clinical practice, as noted in the ICMR guidelines for breast cancer treatment.

CAB has been developed and validated on Indian patients and have been used in clinical settings since the last 3 years. Prescriptions were higher for patients with luminal B, node-negative patients where the physician would want a second opinion to spare the patient from chemotherapy, who would have otherwise got it as per the St. Gallen’s recommendations and other free online tools. Based on the feedback from these treating physicians, 92% of the low-risk patients were spared chemotherapy. Eighty-eight percent of patients were given or not given chemotherapy depending on whether they were CAB high or low risk respectively, indicating a reasonable physician acceptance of CAB as a test to effectively plan treatment decisions. Considering that CAB is not a stand-alone test to decide treatment decision, it is interesting to note that we observed a higher percentage of the low-risk group not receiving chemotherapy (92%) than the high-risk group receiving chemotherapy (80%). This indicates a paradigm shift in the clinical management of the disease, with an intention to decrease the use of chemotherapy in treatment of early-stage patients. With all prescriptions for the test coming in with the need to treat patients with chemotherapy, the change in decision from chemoendocrine therapy treatment to endocrine therapy alone in 92% of patients is very encouraging compared with the rates of 20–50% reported for other tests like ODX, MammaPrint, and EndoPredict [30–34]. Though we did not observe any recurrence in the low-risk patients thus far, we understand that the 5-year follow-up period is not yet completed to conclusively comment on the accuracy of prediction of recurrence by CAB. The main objective of this study is to assess impact of CAB on physician’s treatment planning decision. We observed an increase in the adoption of the CAB by fourfold since 2016 when the test was first launched. All of these patients tested by CAB would be followed up for 5 years to correlate risk prediction to outcome and to determine the accuracy of risk prediction.

In conclusion, we observed a steady increase in the adoption of CAB by physicians and its use for tailoring treatment of patients. CAB provides affordable, accurate alternative prognostic test helping up to 70% patients avoid chemotherapy.
